# Construction of three-dimensional temperature distribution using a network of ultrasonic transducers

**DOI:** 10.1038/s41598-019-49088-y

**Published:** 2019-09-04

**Authors:** Xuehua Shen, Huanting Chen, Tien-Mo Shih, Qingyu Xiong, Hualin Zhang

**Affiliations:** 1 Schoolof Physics and Information Engineering, Minnan Normal University, Zhangzhou, 363000 China; 2 Department of Mechanical Engineering, University of California, Berkeley, CA 94720 USA; 3 School of Software Engineering, Chongqing University, Chongqing, 400044 China

**Keywords:** Applied physics, Techniques and instrumentation

## Abstract

Although the ultrasonic technique for measuring temperature distributions has drawn much attention in recent years, most studies that adopt this technique focus on two-dimensional (2D) systems. Mathematically, extending from 2D to 3D requires higher construction-performing algorithms, as well as more complicated, but extremely crucial, designs of ultrasonic transducer layouts. Otherwise the ill condition of governing-equation matrices will become more serious. Here, we aim at constructing 3D temperature distributions by using a network of properly-installed ultrasonic transducers that can be controlled to transmit and receive ultrasound. In addition, the proposed method is capable of performing this construction procedure in real time, thus monitoring transient temperature distributions and guarantee the safety of operations related to heating or burning. Numerical simulations include constructions for four kinds of temperature distributions, as well as corresponding qualitative and quantitative analyses. Finally, our study offers a guide in developing non-intrusive experimental methods that measure 3D temperature distributions in real time.

## Introduction

Ultrasonic techniques for non-intrusive measurements have been used in both the academia and the industry^1–19^. Particularly, in reference^3^, a method of ultrasonic scattering measurement is presented for nondestructive testing of additively manufactured material, which can avoid both missed detections and false positives. Due to temperatures’ characteristics of spatial distributing and time-varying, the ultrasonic method has drawn much attention in areas of heat transfer and thermometry in recent years^6–11^. Particularly, by ultrasonic pulse-echo measurements, the inverse ultrasonic technique raised in reference^8^ can be used for high-accurately determining internal transient temperature distributions in heat materials. According to references^9–12^, the ultrasound velocity varies dependently on temperature. Particularly, a one-to-one relationship exists between the ultrasound velocity and the temperature, allowing the temperature to be computed after the ultrasound velocity is acquired^13,14^. Conversely, within a given 3D space, the temperature distribution may be constructed if the distribution of ultrasound velocity is acquired by some means^15^.

In the proposed study, we focus on constructing 3D temperature distributions using a network of properly-installed ultrasonic transducers that can be controlled to transmit and receive ultrasounds in different times. Under such circumstances, ultrasound signals will propagate among ultrasonic transducers and will be distributed within the 3D space. In fact, constructing temperature distributions based on ultrasound signals constitutes an inversion process generally accompanied by ill-conditioned matrices^9^. Therefore, mathematical methods of Multi-quadric radial basis approximation and singular-value decomposition are adopted, incorporating advantages of fitting sparse data and solving inversion problems^16^. To validate the constructing performance, we carry out a series of numerical simulations in which temperature-distribution models with different complexity levels are designed. Qualitative and quantitative analyses of simulations theoretically indicate that this ultrasonic method can construct 3D temperature distributions with adequate accuracy, strong anti-interference and satisfactory real-time capability.

## Description of Temperature Distribution Construction

As mentioned above, the ultrasound velocity changes depending on temperatures. In gases, the relationship between ultrasound velocity and temperatures is described as^6,17,18^

1 $$v=\sqrt{\kappa \frac{R}{{M}_{g}}T}=B\sqrt{T},$$

where *v* denotes the ultrasound velocity; *T* the absolute temperature; *R* the universal gas constant; *κ* and *M*_*g*_ respectively the adiabatic exponent and the average molecular weight of the gas. As constants, *κ*, *M*_*g*_ and *R* can be grouped and replaced by another constant *B*.

**Figure 1:**
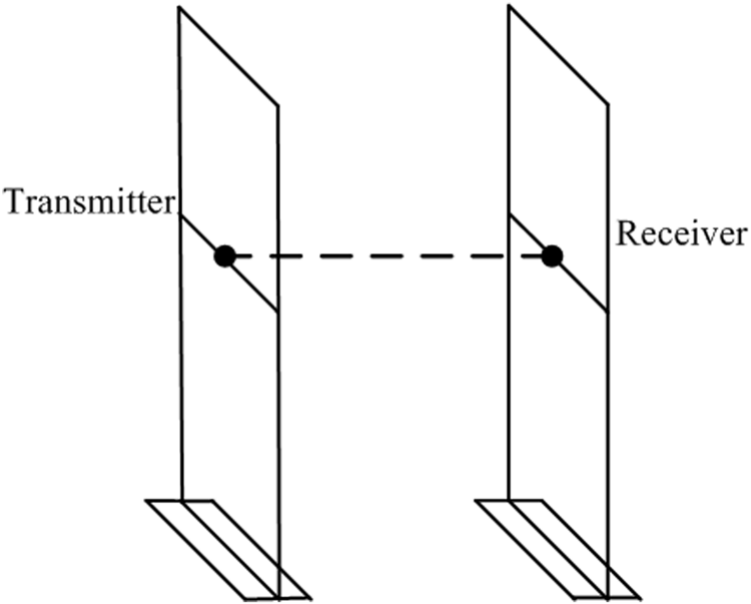
Illustration of single-path ultrasonic method.

For situations with uniform temperatures, the simplest device illustrated in Fig. 1 will meet most demands. This device is composed of an ultrasonic transmitter and an ultrasonic receiver. Since distance *L* between the transmitter and the receiver is fixed, we can compute the uniform temperature by

2 $$T={(L/t)}^{2}/{B}^{2},$$

if *t* is measured.

However, in various realistic situations with non-uniform temperatures, average temperatures no longer suffice. For constructing 3D temperature distributions, numerous ultrasonic transducers should be installed spatially to ensure adequate precision.

**Figure 2:**
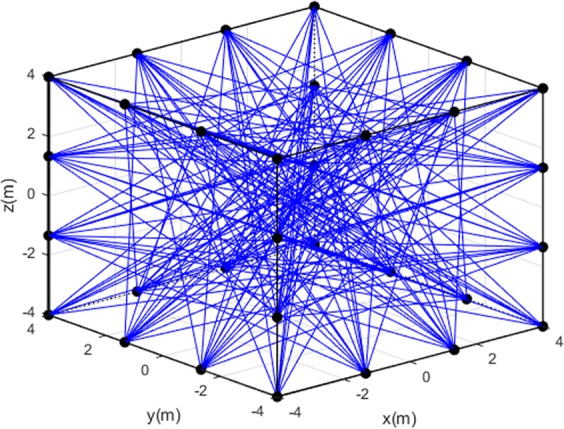
The network of ultrasonic transducer and ultrasound paths.

A network of 32 ultrasonic transducers is represented by black dots and installed uniformly on each edge of the cube (Fig. 2). These transducers can be regarded as transceivers due to their ability of working as transmitters and receivers, which are controlled to transmit and detect ultrasonic signals in different times^16^. Ultrasound signals propagate from one transducer to another transducer and generate ultrasound paths, which are represented by blue lines. Theoretically, ultrasound paths exist between every two transducers^17^. However, since ultrasound paths on edges or surfaces contribute little to the construction of internal temperatures, they are non-essential and have been eliminated from Fig. 2. Totally, these 32 ultrasonic transducers generate 172 effective ultrasound paths. We obtain

3 $${t}_{k}=\mathop{\int }\limits_{{l}_{k}}\,1/v(x,y,z)\,dl=\mathop{\int }\limits_{{l}_{k}}\,f(x,y,z)\,dl,$$

where *l*_*k*_ denotes the *k*^*th*^ ultrasound path, *t*_*k*_ travelling time of the ultrasound path *l*_*k*_, and *f*(*x*, *y*, *z*) the reciprocal of *v*.

**Figure 3:**
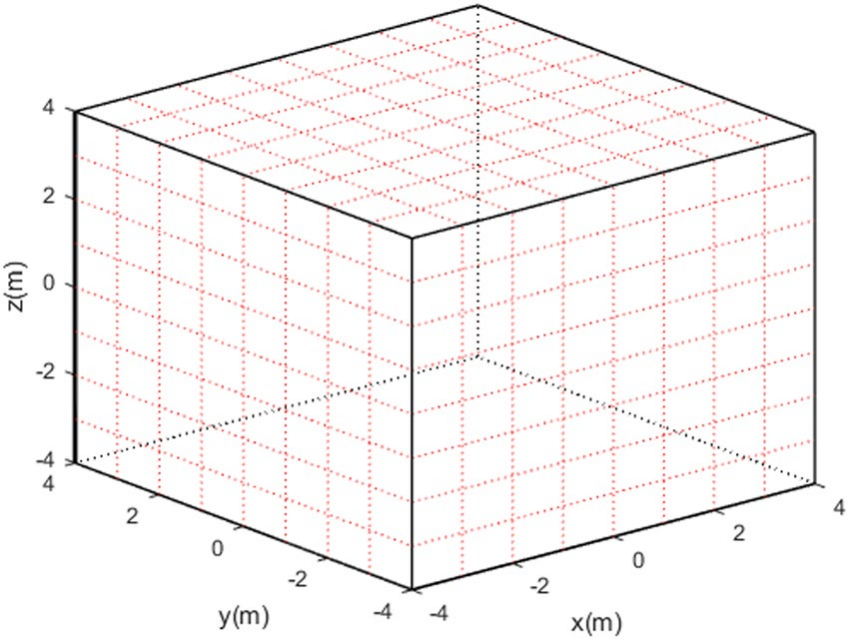
Dividing of the cubic area.

The process of constructing temperature distributions involves Multi-quadric radial basis approximation. To proceed, we divide the measured area into *N* elements, and then establish *N* radial basis functions whose centers correspond to centers of those elements (Fig. 3, *N* = 512 = 8 × 8 × 8).

The Multi-quadric radial basis function is established as

4 $${\phi }_{i}(x,y,z)=\sqrt{\parallel (x,y,z)-({x}_{i},{y}_{i},{z}_{i}){\parallel }^{2}+{\gamma }^{2}},$$

where ||·|| denotes the standard Euclidean norm; (*x*_*i*_, *y*_*i*_, *z*_*i*_) the center of Multi-quadric radial basis, or the midpoint of the *i*^*th*^ element; and *γ* the smooth factor related to the specific area as well as the network of transducers^16^.

Next, *f*(*x*, *y*, *z*) is expressed as a linear combination of *N* Multi-quadric radial basis functions:

5 $$f(x,y,z)=\mathop{\sum }\limits_{i=1}^{N}\,{\varepsilon }_{i}\cdot {\phi }_{i}(x,y,z),$$

where *ε*_*i*_ is an undetermined coefficient describing the distribution of ultrasound velocity’s reciprocal.

Define *a*_*ki*_ as:

6 $${a}_{ki}=\mathop{\int }\limits_{{l}_{k}}\,{\phi }_{i}(x,y,z)\,dl=\mathop{\int }\limits_{{l}_{k}}\,\sqrt{\parallel (x,y,z)-({x}_{i},{y}_{i},{z}_{i}){\parallel }^{2}+{\gamma }^{2}}\,dl.$$

To compute coefficient *ε*_*i*_, we can merge Eqs (3)–(6) to obtain

7 $${t}_{k}=\mathop{\sum }\limits_{i=1}^{N}\,{\varepsilon }_{i}\,\mathop{\int }\limits_{{l}_{k}}\,{\phi }_{i}(x,y,z)\,dl=\mathop{\sum }\limits_{i=1}^{N}\,{a}_{ki}\,{\varepsilon }_{i}.$$

With definitions given as

8 $$\begin{array}{rcl}A & = & ({a}_{ki}),\,k=1,2,\ldots ,M,\,i=1,2,\ldots ,N,\\ \varepsilon  & = & {({\varepsilon }_{1},{\varepsilon }_{2},\ldots ,{\varepsilon }_{N})}^{T},\\ t & = & {({t}_{1},{t}_{2},\ldots ,{t}_{M})}^{T},\end{array}$$

we can rewrite Eq. (7) into a matrix form as

9 $$t=A\varepsilon .$$

Considering *A* as an ill-conditioned matrix, we adopt the singular-value decomposition to achieve regularization estimation. According to matrix theory^20^, any real matrix $A\in {R}^{M\times N}$ can be decomposed as

10 $$\begin{array}{rcl}A & = & US{V}^{T},\\ S & = & [\begin{array}{cc}\sum  & 0\\ 0 & 0\end{array}],\,S\in {R}^{M\times N},\\ \sum  & = & diag({\sigma }_{1},{\sigma }_{2},\ldots ,{\sigma }_{r}),\end{array}$$

where $U\in {R}^{M\times M}$ and $V\in {R}^{N\times N}$ denote two orthogonal matrices; *r* the rank of matrix *A*; and ${\sigma }_{1}\ge {\sigma }_{2}\ge \cdots \ge {\sigma }_{r} > 0$ the *r* singular values of matrix *A*. Let us further define

11 $$\begin{array}{rcl}{S}^{+} & = & [\begin{array}{cc}{\sum }^{-1} & 0\\ 0 & 0\end{array}],\,{S}^{+}\in {R}^{N\times M},\\ {\sum }^{-1} & = & diag(1/{\sigma }_{1},1/{\sigma }_{2},\ldots ,1/{\sigma }_{r}),\end{array}$$

then the generalized inverse matrix of *A*_,_ i.e.${A}^{+}\in {R}^{N\times M}$ is obtained as

12 $${A}^{+}=V{S}^{+}{U}^{T},$$

Thus, the undetermined coefficient *ε* can be rewritten as

13 $$\varepsilon ={A}^{+}t.$$

Since parameter *γ* is determined appropriately via the numerical method, elements of matrix *A* can be computed in advance to predetermine *A*^+^. When matrix of travelling times *t* are measured, undetermined coefficients *ε* will be obtained. Thus, the reciprocal of ultrasound velocity *f*(*x*, *y*, *z*) and then ultrasound velocity *v*(*x*, *y*, *z*) can be eventually determined. Finally, the temperature distribution *T*(*x*, *y*, *z*) is constructed as

14 $$T(x,y,z)=\frac{1}{{B}^{2}}\cdot {v}^{2}(x,y,z)=1/{[B\mathop{\sum }\limits_{i=1}^{N}{\varepsilon }_{i}\cdot {\phi }_{i}(x,y,z)]}^{2}{\rm{.}}$$

By the expression *T*(*x*, *y*, *z*), not only the temperature of any position with exact coordinates, but also the temperature distribution of the whole measured area can be constructed.

## Numerical Simulations and Analysis

To validate the temperature distribution construction performance of this ultrasonic method, we carry out a series of numerical simulations. As shown in Figs 2 and 3, the measured area is a cube of 8*m* × 8*m* × 8*m*, with 32 ultrasonic transducers generating 172 effective ultrasound paths. The cube is divided into 512 uniform elements to establish 512 Multi-quadric radial basis functions. In addition, constant coefficient *B* in gaseous environment is 20.03^18,19^, and parameters *γ* is predetermined as 0.1. In simulations, four temperature distribution models with different complexity levels are set up to serve as references to constructions below.

One-peak symmetrical temperature distribution:

15 $${T}_{1}(x,y,z)=\frac{1800}{0.5\times ({x}^{2}+{y}^{2}+{z}^{2}+1)},$$

One-peak asymmetrical temperature distribution:

16 $${T}_{2}(x,y,z)=\frac{1800}{0.5\times [{(x+2)}^{2}+{(y+2)}^{2}+{z}^{2}+1]},$$

Two-peak temperature distribution:

17 $$\begin{array}{rcl}{T}_{3}(x,y,z) & = & \frac{1500}{1.5\times [{(x+1.8)}^{2}+{(y+1.8)}^{2}+{(z-2)}^{2}+1]}\\  &  & +\,\frac{1500}{1.5\times [{(x-1.8)}^{2}+{(y-1.8)}^{2}+{(z-2)}^{2}+1]},\end{array}$$

Four-peak temperature distribution:

18 $$\begin{array}{rcl}{T}_{4}(x,y,z) & = & \frac{1200}{2\times [{(x+1.8)}^{2}+{(y+1.8)}^{2}+{(z-2)}^{2}+1]}\\  &  & +\,\frac{1200}{2\times [{(x+1.8)}^{2}+{(y-1.8)}^{2}+{(z+2)}^{2}+1]}\\  &  & +\,\frac{1200}{2\times [{(x-1.8)}^{2}+{(y-1.8)}^{2}+{(z+2)}^{2}+1]}\\  &  & +\,\frac{1200}{2\times [{(x-1.8)}^{2}+{(y-1.8)}^{2}+{(z-2)}^{2}+1]}{\rm{.}}\end{array}$$

Known from practical experiences, travelling times of ultrasound paths obtained via actual measurements may be influenced by various interferences^21–23^. Thus, to investigate the anti-interference of this ultrasonic method, we implement experiments respectively in both ideal condition and non-ideal condition. In the former, travelling times are theoretically computed from Eq. (19). In the latter, travelling times are assumed to be theoretical values over laid with Gaussian noise, whose mean deviation and standard deviation are 0 and 0.001 respectively.

19 $${t}_{k}=\mathop{\int }\limits_{{l}_{k}}\,\frac{1}{B\sqrt{{T}_{i}(x,y,z)}}dl,\,i=1,2,3,4.$$

To make comprehensive evaluations, we analyze simulations qualitatively and quantitatively. The former includes slice maps and scatter plots of temperature distributions, the latter consists of temperature mean relative error *E*_*m*_, temperature root-mean-square percent error *E*_*r*_, as well as average construction time. Here, *E*_*m*_ and *E*_*r*_ are respectively defined as:

20 $${E}_{m}=\frac{1}{n}\,\mathop{\sum }\limits_{j=1}^{n}\,|T{R}_{j}-T{M}_{j}|,$$

and

21 $${E}_{r}=\frac{\sqrt{\frac{1}{n}\,{\sum }_{j=1}^{n}\,{|T{R}_{j}-T{M}_{j}|}^{2}}}{T{M}_{m}}\times 100 \% ,$$

where *n* denotes the number of computed points; *TR*_*j*_ and *TM*_*j*_ temperatures of point (*x*_*j*_, *y*_j_, *z*_*j*_) respectively of the construction and its model; and *TM*_*m*_ the mean temperature of model.

**Figure 4:**
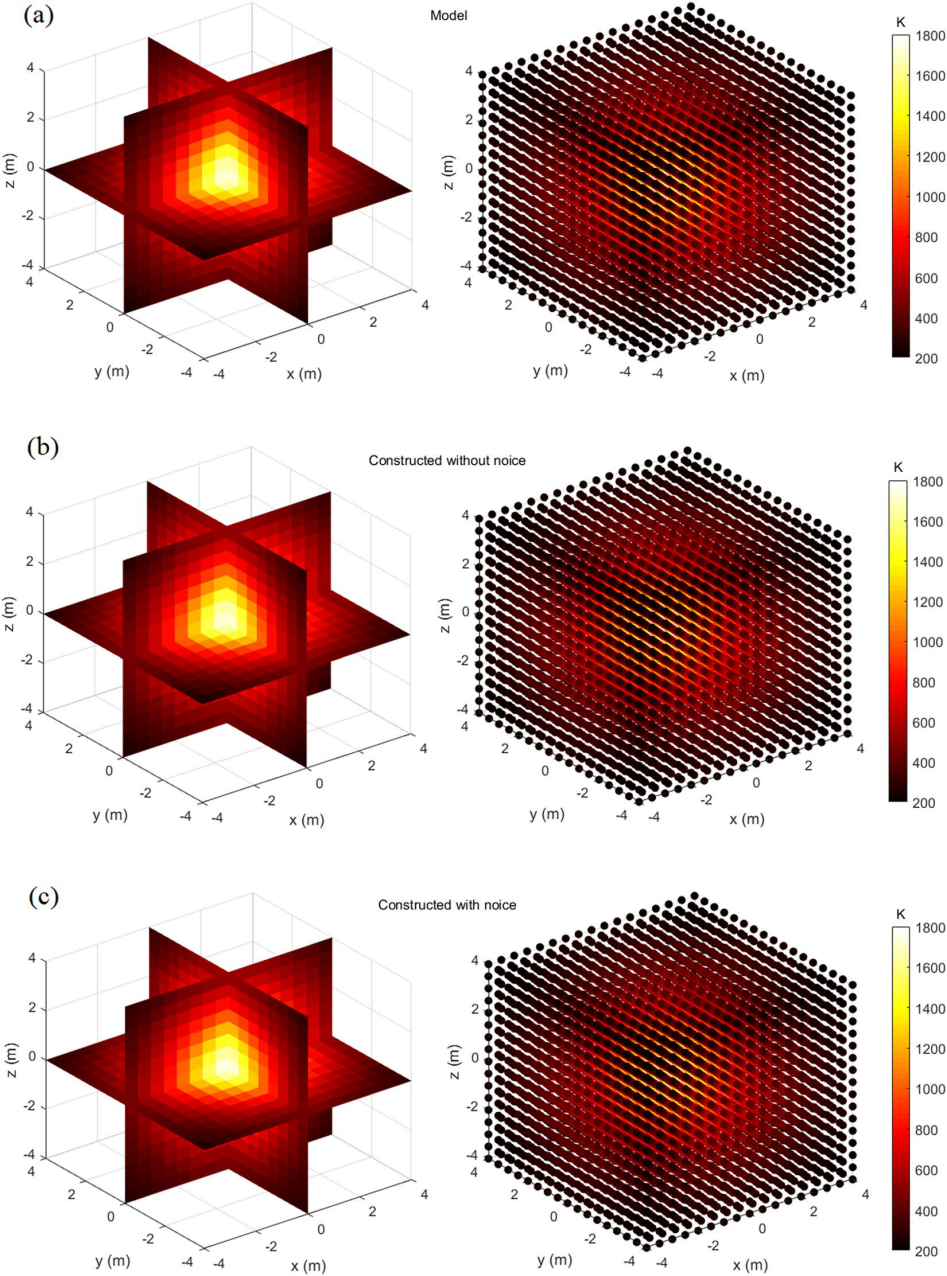
One-peak symmetrical temperature distribution model and its constructions, the left one is slice map of temperature distribution, and the right one is scatter plot of temperature distribution: (**a**) Model; (**b**) Construction without noise; (**c**) Construction with noise.

**Figure 5:**
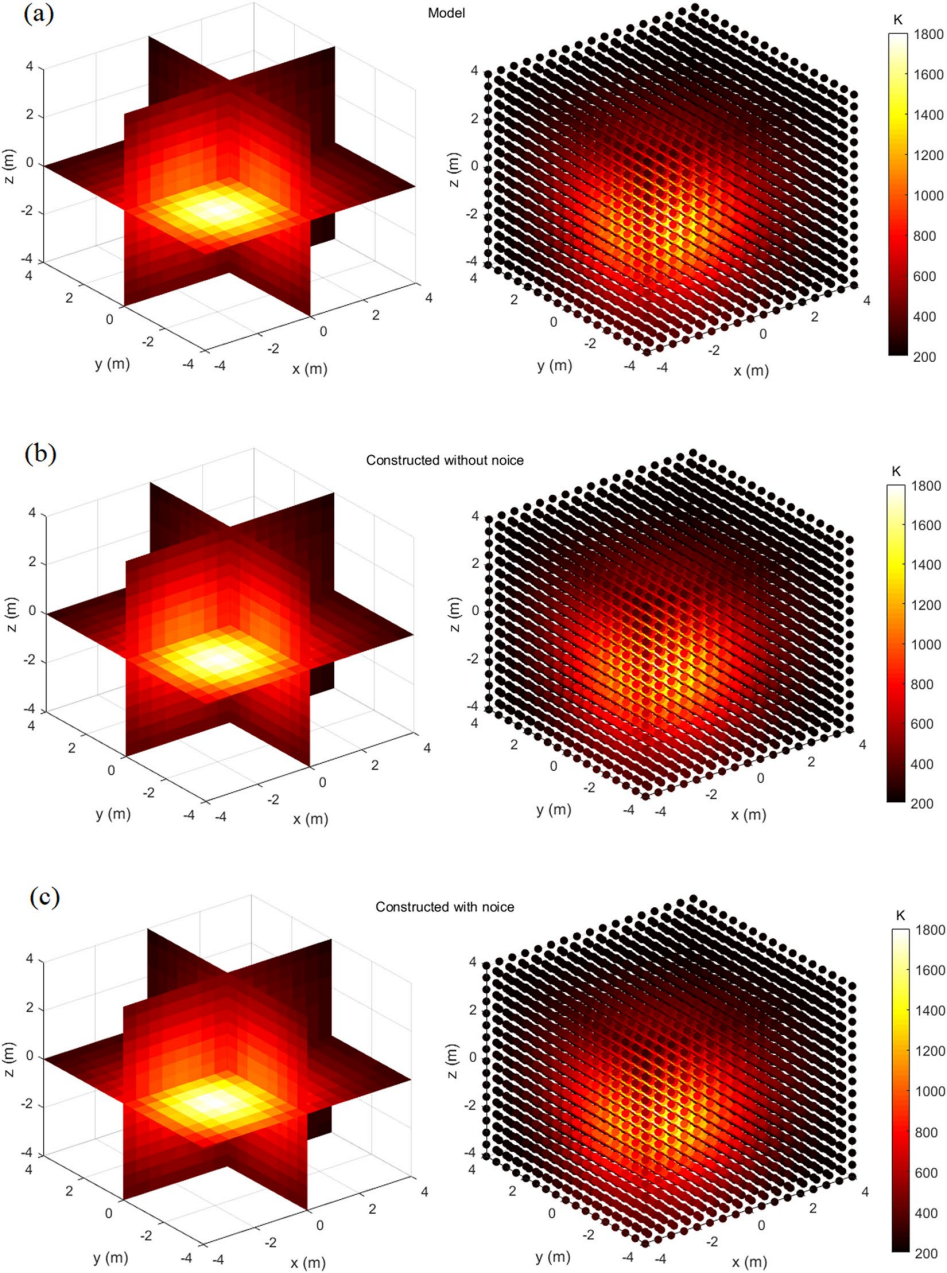
One-peak asymmetrical temperature distribution model and its constructions, the left one is slice map of temperature distribution, and the right one is scatter plot of temperature distributions: (**a**) Model; (**b**) Construction without noise; (**c**) Construction with noise.

**Figure 6:**
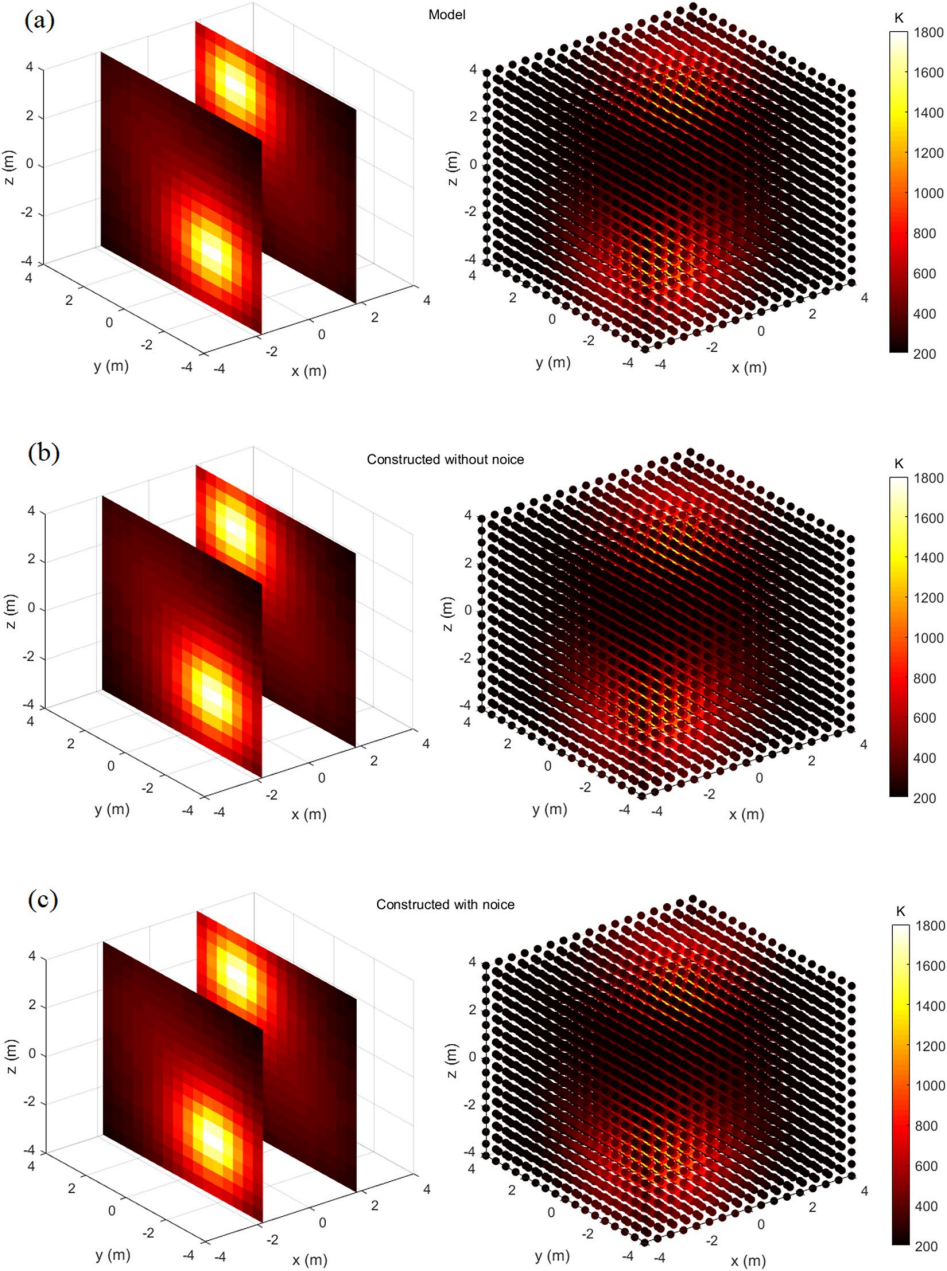
Two-peak temperature distribution model and its constructions, the left one is slice map of temperature distribution, and the right one is scatter plot of temperature distribution: (**a**) Model; (**b**) Construction without noise; (**c**) Construction with noise.

**Figure 7:**
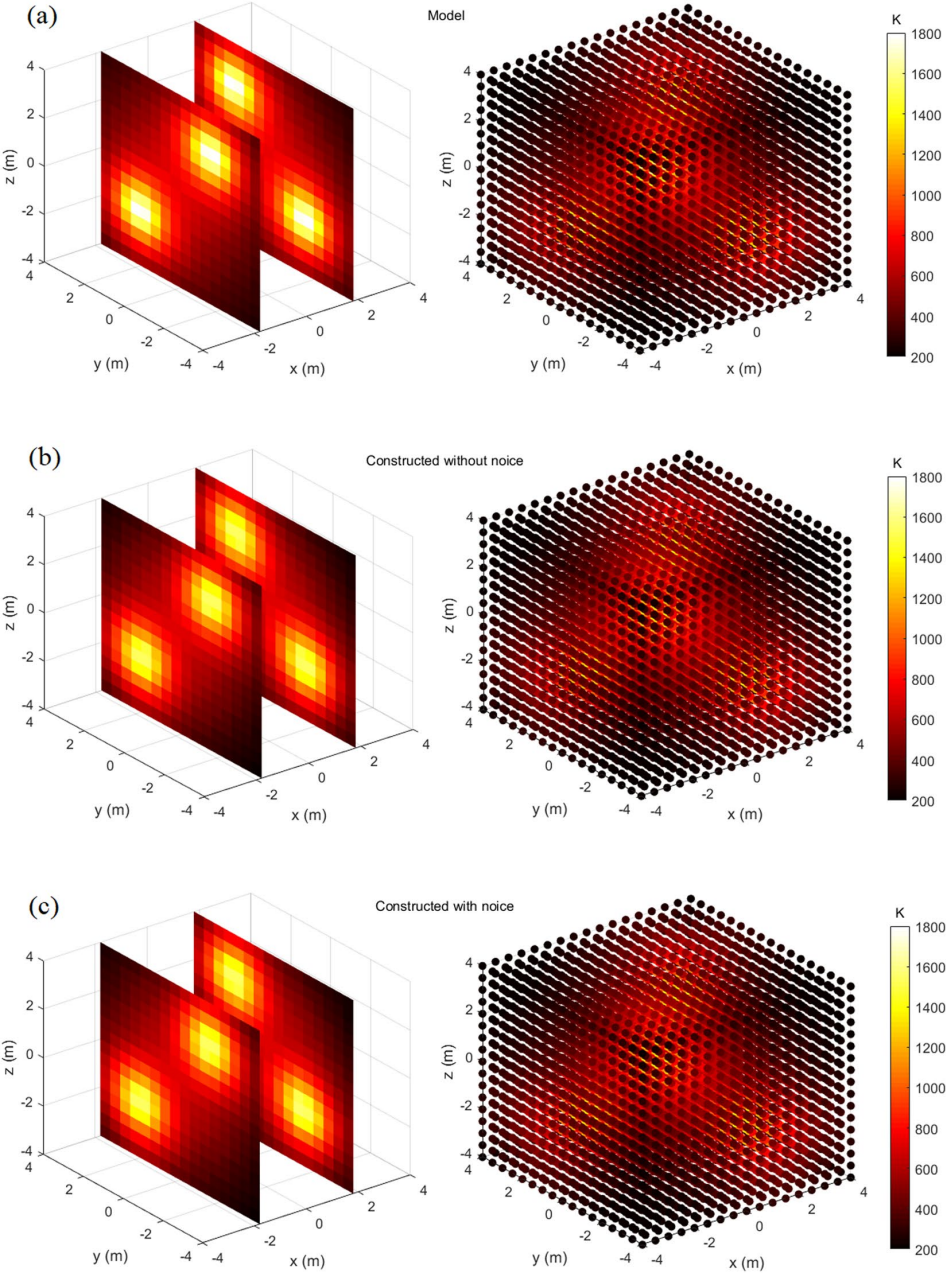
Four-peak temperature distribution model and its constructions, the left one is slice map of temperature distribution, and the right one is scatter plot of temperature distribution: (**a**) Model; (**b**) Construction without noise; (**c**) Construction with noise.

Figures 4–7 display the slice map and scatter plot of temperature distributions, respectively of models and their constructions. As figures show, these constructions resemble, and almost look identical to, their corresponding models. Even for the most complex four-peak temperature distribution and under the influence of noise, it is difficult to distinguish constructions from the model via the visual inspection.

Quantitative temperature errors and construction times are computed and respectively displayed in Tables 1 and 2, which can further evaluate the construction performance. Considering impact of random factors, simulations that construct a certain temperature distribution model are repeatedly done 1000 times. Thus, these quantitative temperature errors and construction times are average values.

**Table 1:** Quantitative temperature errors of constructions (%).

Models	Without noise		With noise	
	*E*_*m*_	*E*_*r*_	*E*_*m*_	*E*_*r*_
*T*_*1*_ (*x*, *y*)	0.281	0.115	3.103	0.956
*T*_*2*_ (*x*, *y*)	0.602	0.319	2.977	1.187
*T*_*3*_ (*x*, *y*)	1.640	1.067	3.233	1.651
*T*_*4*_ (*x*, *y*)	7.854	3.965	8.680	4.212

**Table 2:** Average reconstruction times (s).

Models	Average reconstruction time
*T*_*1*_ (*x*, *y*)	0.0837
*T*_*2*_ (*x*, *y*)	0.0921
*T*_*3*_ (*x*, *y*)	0.0943
*T*_*4*_ (*x*, *y*)	0.1008

Observing from Table 1, we find out that errors in constructions of complex two-peak and four-peak temperature distributions are relatively larger than those of simple ones. And for a specific temperature distribution, errors under influence of noise are slightly larger than those without influence of noise. On the whole, constructions in various situations are accurate enough, as *E*_*m*_ and *E*_*r*_ are respectively within 8.7% and 4.3%.

Conclusions can be drawn from qualitative error figures and quantitative errors. First, although constructions may be accompanied by some errors, they can accurately describe how temperatures distribute over the whole area, especially for hot peaks. Second, construction errors have tendency to increase with complexity of temperature distributions and influences of noise.

To some extent, average construction times demonstrate the real-time capability of this ultrasonic method (Table 2). Clearly, the proposed method can construct various temperature distributions within about 0.089s–0.102s and provide real-time temperature distributions in most situations.

## Conclusion

Based on the one-to-one relationship between the ultrasound velocity and the temperature, we have developed a non-intrusive and real-time ultrasonic technique that can compute 3D temperature distributions using a network of properly-installed ultrasonic transducers. Due to the ill-posed condition in the process, multi-quadric radial basis approximation and singular value decomposition are adopted, incorporating advantages of fitting sparse data and solving inversions. To validate the constructing performance, we further carry out a series of numerical simulations in which temperature distribution models with different complexity levels are designed.

This paper performed numerical simulations but not experimental validation is in three considerations. First, this study focuses on the theoretical feasibility of constructing 3D temperature distributions using ultrasonic technique, and numerical simulations are convincing enough. Second, the experimental validation requires absolutely accurate realistic temperature distributions for references, but it is difficult and even impossible to accurately measure realistic temperature distributions using existing thermometry techniques. Third, inaccurate travelling times of ultrasound paths may cause unsatisfactory constructed temperature distributions, whether the construction algorithm is excellent enough or not. Therefore, in realistic situations, the measured travelling times of ultrasound paths frequently affected by uncertain interferences may lead to an incorrect assessment of the ultrasonic technique in temperature distribution constructions.

Since this paper has demonstrated the theoretical feasibility of the ultrasonic technique in constructing 3D temperature distributions, our future studies will focus on its experimental validation and engineering application, including the selection of hardware components, the design and establishment of experimental platform, the performance optimization of ultrasonic transducers, the processing, extraction and transformation of ultrasonic signals, etc. The main factors affect the accuracy of realistic temperature distribution constructions may lie in the uniformity of the measured media, the flow of gas, the size and layout of ultrasonic transducers, and the time delay in transmitting and receiving of ultrasound signals, etc., and these therefore must be emphases in our future studies.

## Data Availability

The datasets generated and analyzed during the current study are available from the corresponding author on reasonable request.
